# Differential Exchange of Multifunctional Liposomes Between Glioblastoma Cells and Healthy Astrocytes via Tunneling Nanotubes

**DOI:** 10.3389/fbioe.2019.00403

**Published:** 2019-12-12

**Authors:** Beatrice Formicola, Alessia D'Aloia, Roberta Dal Magro, Simone Stucchi, Roberta Rigolio, Michela Ceriani, Francesca Re

**Affiliations:** ^1^School of Medicine and Surgery, University of Milano-Bicocca, Vedano al Lambro, Italy; ^2^Department of Biotechnology and Biosciences, University of Milano-Bicocca, Milan, Italy

**Keywords:** glioblastoma, liposomes, tunneling nanotubes, doxorubicin, nanoparticles, nanomedicine

## Abstract

Despite advances in cancer therapies, nanomedicine approaches including the treatment of glioblastoma (GBM), the most common, aggressive brain tumor, remains inefficient. These failures are likely attributable to the complex and not yet completely known biology of this tumor, which is responsible for its strong invasiveness, high degree of metastasis, high proliferation potential, and resistance to radiation and chemotherapy. The intimate connection through which the cells communicate between them plays an important role in these biological processes. In this scenario, tunneling nanotubes (TnTs) are recently gaining importance as a key feature in tumor progression and in particular in the re-growth of GBM after surgery. In this context, we firstly identified structural differences of TnTs formed by U87-MG cells, as model of GBM cells, in comparison with those formed by normal human astrocytes (NHA), used as a model of healthy cells. Successively, we have studied the possibility to exploit U87-MG TnTs as drug-delivery channels in cancer therapy, using liposomes composed of cholesterol/sphingomyelin and surface functionalized with mApoE and chlorotoxin peptides (Mf-LIP) as nanovehicle model. The results showed that U87-MG cells formed almost exclusively thick and long protrusions, whereas NHA formed more thin and short TnTs. Considering that thick TnTs are more efficient in transport of vesicles and organelles, we showed that fluorescent-labeled Mf-LIP can be transported via TnTs between U87-MG cells and with less extent through the protrusions formed by NHA cells. Our results demonstrate that nanotubes are potentially useful as drug-delivery channels for cancer therapy, facilitating the intercellular redistribution of this drug in close and far away cells, thus reaching isolated tumor niches that are hardly targeted by simple drug diffusion in the brain parenchyma. Moreover, the differences identified in TnTs formed by GBM and NHA cells can be exploited to increase treatment precision and specificity.

## Introduction

The limits of conventional therapies against tumors, in terms of effectiveness/damage ratio, lead to the development and application in clinics of different nanotechnological drugs in the last 25 years (Stupp et al., 2009). Many advancements have been achieved in this field, but different issues, such as the complexities and heterogeneity of tumor biology, still remain unsolved. Gliomas, intrinsic brain tumors, are a dissimilar group of oncological diseases for which there is currently no cure, and only very limited progress has been made in the control of the disease course over the past three decades (Westphal and Lamszus, 2011). Among gliomas, glioblastoma multiforme (GBM, also called grade IV astrocytoma) is one of the most deadly brain tumors, with a short median patient survival and a very limited response to therapies (Louis et al., 2016). In this context, many efforts are underway toward the development of new therapeutic approaches and nanomedicine seems to be one of the most promising. Nevertheless, many obstacles have not been overcome yet. GBM has a very complex pathogenesis that involves alterations of several key cellular pathways, diffuse invasiveness, and capacity to escape therapies. An important component of tumor growth is communication within cancer cells and with other cells in the microenvironments, which strengthen tumor progression and resistance to radiotherapy and chemotherapy (Broekman et al., 2018).

Normal and tumor cells exploit different communication modalities, and one of them is represented by the physical connection via tunneling nanotubes (TnTs) and microtubes (TmTs), which form a cytoplasmic continuum between cells and allow the transport of non-secretable molecules and organelles. In particular, TnTs can mediate the transfer of cellular vesicles (Rustom et al., 2004; Önfelt et al., 2006), mitochondria (Ahmad et al., 2014), lysosomes (Abounit et al., 2016), miRNAs (Thayanithy et al., 2014), single proteins (Schiller et al., 2013), and viral particles (Sowinski et al., 2008) between cells, also very distant from each other (>100 μm of distance). TnTs are transient transcellular channels with a diameter of 50–200 nm, a length up to several cell diameters with variable lifetimes ranging from <60 min up to many hours (Carone et al., 2015).

Lou et al. (2012a,b) firstly described the presence of TnTs in human primary tumors and in many cancer cell lines, highlighting the key role of these membranous structures in cancer cell pathogenesis and invasion. The involvement of TnTs and TmTs has also been indicated in the re-growth of GBM after surgery and in conferring resistance to chemotherapy (Moschoi et al., 2016; Weil et al., 2017). Although TnTs are not apparent in some glioma cellular models (Van der Vos et al., 2016), they may represent a new tool for bidirectional intercellular transfer of drug-loaded nanoparticles.

In this context, there are some data supporting the direct cell-to-cell transfer of nanoparticles through TnTs, and this strategy may be exploited to increase the range of drug delivery between cancer cells (Epperla et al., 2015; Sisakhtnezhad and Khosravi, 2015; Deng et al., 2018). One of the peculiarity of GBM is the presence of glioma stem cells both within the tumor bulk, which are able to reconstitute a whole tumor after surgical resection (Fan et al., 2010; Lin et al., 2010), and in other brain regions, where minor stem-cell niches represent a pool from which new tumor cells originate (Gould, 2017). Then, targeting primary GBM with nanotherapeutics may allow the possibility to reach via TnTs isolated, infiltrating tumor cells (stem cells included) that are hardly reached by drug diffusion in the brain parenchyma.

This study aims to evaluate *in vitro* the possible intercellular transport of multifunctional liposomes (LIP) via TnTs between human primary glioblastoma cell line. We have recently designed LIP carrying doxorubicin, as an anti-cancer drug model, and dually functionalized with apoE-derived peptide and with chlorotoxin (ClTx), as GBM targeting ligands (DeBin et al., 1993; Maletínská et al., 2000; Lyons et al., 2002; Xiang et al., 2011; Ojeda et al., 2016). The ability of LIP functionalized with apoE-derived peptide (namely, mApoE) to cross the blood–brain barrier both *in vitro* and *in vivo*, was already reported (Re et al., 2011; Balducci et al., 2014; Bana et al., 2014; Dal Magro et al., 2018).

LIP trafficking via TnTs in GBM cells has not been reported yet. Moreover, we compared the heterogeneity of TnTs, in terms of structure, morphology, size, and abundance between GBM cells and human healthy astrocytes, with the aim to increase the precision and specificity of treatments.

## Materials and Methods

### Materials

Cholesterol (Chol), doxorubicin (DOX), thiazolyl blue tetrazolium bromide, 4-(2-hydroxyethyl)piperazine-1-ethanesulfonic acid (HEPES), Triton X-100, ultra-low-range molecular weight marker (MW 1,060–26,600), EZBlue Gel Staining Reagent, 1,1′-dioctadecyl-3,3,3′,3′-tetramethylindocarbocyanine perchlorate (DiI probe), TRITC-phalloidin, and mouse monoclonal anti-β-tubulin antibody were purchased from Sigma Aldrich (Milan, Italy). 1,2-Distearoyl-*sn*-glycero-3-phospho-ethanolamine-*N*[maleimide(polyethyleneglycol)-2000] (mal-PEG-DSPE) and sphingomyelin from bovine brain (Sm) were purchased from Avanti Polar Lipids, Inc. (Alabaster, AL, USA). BODIPY™ FL C 12-sphingomyelin (BODIPY-Sm) was purchased from Thermo Fisher Scientific. 1,2-Distearoyl-*sn*-glycero-3-phospho-ethanolamine-*N*[(polyethyleneglycol)-2000] *n*-hydroxysuccinimide ester (NHS-PEG-DSPE) was purchased from Nanocs (Boston, USA). Ultrapure and deionized water was obtained from Direct-Q5 system (Millipore, Italy). mApoE peptide (CWG-LRKLRKRLLR, MW 1,698.18 g/mol) and ClTx (MW 4,004 g/mol) were synthetized by KareBay Biochem (Monmouth Junction, NJ, USA). Dialysis membranes (cutoff 12,000–14,000 Da) were purchased from Medicell International Ltd (London, UK). Penicillin–streptomycin (P/S) solution 100× was purchased from Euroclone (Milan, Italy); phosphate-buffered saline (PBS) 1×, collagen, trypsin/EDTA solution, and NuPAGE Bis-Tris (4–12%) were from Invitrogen. All other chemicals were of analytical grade and were obtained from either Sigma Aldrich or Merck. Alexa Fluor 488 goat anti-mouse IgG and CellTrace Far Red Dye (CT) were from Life Technologies. 3,3′-Dioctadecyloxacarbocyanine perchlorate (DiO) was from Sigma Aldrich (Milan, Italy).

### Preparation of Chlorotoxin–PEG-DSPE

ClTx-lipid was prepared as described in Xiang et al. (2011) with small modifications. Briefly, 0.1 μmol of NHS-PEG-DSPE in CHCl_3_/MeOH (2:1, vol/vol) was dried under N_2_ to remove organic solvents. Then 5 eq (0.5 μmol) of ClTx dissolved in 10 mM of Hepes and 150 mM of NaCl pH 7.4 was added to the dried lipid. The reaction was conducted under gentle stirring for 90 min at room temperature. The resulting solution was dialyzed against MilliQ water for 2 days in a dialysis tube (molecular weight cutoff [MWCO] = 12–14,000 Da) to remove unreacted ClTx and then lyophilized overnight.

### Preparation Multifunctional Liposomes

LIP were composed of sphingomyelin, cholesterol (1:1, mol/mol) added with 2.5 mol% of mal-PEG-PE, for the covalent binding of mApoE peptide, and with 0.5 mol% of BODIPY-Sm as fluorescent probe (Re et al., 2010, 2011). LIP were prepared in 10 mM of Hepes and 150 mM of NaCl pH 7.4 by extrusion procedure through polycarbonate membranes of 100-nm-diameter pores, under N_2_. mApoE peptide was covalently attached on LIP surface by thiol–maleimide coupling, as previously described (Re et al., 2010, 2011). ClTx-lipid was added to mApoE-LIP by post-insertion technique, following the procedure previously described (Mare et al., 2018). This sample will be referred as Mf-LIP. As controls, LIP composed of sphingomyelin and cholesterol (1:1, mol/mol) were prepared in ammonium sulfate (500 mM, pH 5.5) by extrusion procedure as described above. LIP were dialyzed against 10 mM of Hepes and 150 mM of NaCl pH 7.4, overnight, and then incubated with DOX (1.5 μmol of DOX/10 μmol of total lipids) for 1 h at 65°C to allow the incorporation of DOX in the LIP core. This sample will be referred to as DOX-LIP. Mf-LIP and DOX-LIP were purified with a Sepharose G-25 fine column (25 × 1 cm) to remove unbounded and unincorporated materials.

### Characterization of Multifunctional Liposomes

After purification, the amount recovered for each compound was determined by different techniques. Briefly, phospholipids content was quantified by Stewart Assay (Stewart, 1980); the amount of ClTx and mApoE on LIP surface was assessed by sodium dodecyl sulfate–polyacrylamide gel electrophoresis (SDS-PAGE). DOX loading was quantified spectrofluorometrically (λex = 495 nm; λem = 592 nm) after vesicle disruption with 0.1% Triton X-100. The DOX encapsulation yield in LIP was calculated by comparing fluorescence intensities with a previously established calibration curve of free DOX in 10 mM of Hepes and 150 mM of NaCl pH 7.4. Size and polydispersity index (PDI) were analyzed by dynamic light scattering (DLS) technique (Brookhaven Instruments Corporation, Holtsville, NY, USA). ζ-Potential was determined by using an interferometric Doppler velocimetry with the same instrument equipped with ZetaPALS device. LIP stability was measured in 10 mM of Hepes and 150 mM of NaCl pH 7.4 by observing size and PDI by DLS for 1 week.

### Cell Cultures

U87-MG glioblastoma cells were purchased from American Type Culture Collection (ATCC, VA, USA) and were grown in DMEM high glucose, 10% fetal bovine serum (FBS), 1% P/S, and 1% glutamine (Tamborini et al., 2016). Normal human astrocytes (NHA), purchased from Lonza (Walkersville, Maryland, USA), were maintained in astrocyte basal medium supplemented with AGM BulletKit™. All cell lines were cultured at 37°C under a humidified atmosphere containing 5% CO_2_.

### Tunneling Nanotube Analysis by Confocal Microscopy

NHA and U87-MG cells were seeded at a density of 5 × 10^3^ or 1.5 × 10^4^ cells/cm^2^, respectively, on porcine gelatin pretreated coverslips. One day after seeding, cells were treated for 1 or 24 h with free DOX (15 or 25 μg/ml) or with DOX-LIP (15 μg/ml of DOX and 200 nmol of total lipids) or with fluorescent-labeled Mf-LIP (200 nmol of total lipids) at 37°C in 5% CO_2_. Untreated cells were used as a control. After treatment, cells were then left for 2 h in each culture complete medium and then stained for 20 min with 1.9 μl/ml of DiI in PBS (membrane/endocytic vesicles), or with 5 μl/ml of DiO in PBS, to label cell membranes, TnTs included, according to the manufacturer's instructions. Cells were then fixed for 8 min with 3.7% paraformaldehyde in PBS. Fluorescence images were examined with a 40× magnification on A1R Nikon (Nikon, Tokyo, Japan) laser scanning confocal microscope. Cells were carefully scored for the presence of TnTs. About 200 cells for each experiment were analyzed. TnTs were counted. Experiments were performed in triplicate. Images were analyzed by ImageJ software.

### Cellular Uptake of Doxorubicin-Liposomes and Multifunctional Liposomes

U87-MG cells were seeded on a 6-well plate (5 ×10^3^ cells/cm^2^); and after 2 days of culture, cells were treated with free DOX (15 μg/ml) or LIP formulations (DOX 15 μg/ml) for 1 and 3 h. At the two different time points, free DOX and LIP formulations were removed, and the cells were washed with PBS, detached, and treated with lysis buffer (50 mM of Tris–HCl pH 7.4, 150 mM of NaCl, 2 mM of EDTA, 1% Triton X-100, 0.1% SDS, and 1 mM of DTT). Samples were centrifuged at 12,000 rpm for 15 min at 4°C and the DOX fluorescence (λex = 495 nm; λem = 592 nm) in the pellets was measured by Jasco FP-8500 spectrofluorometer. Results were expressed as DOX fluorescence in pellets/DOX fluorescence in initial sample ×100 and indicated as cell uptake (%).

High-throughput images of living U87-MG cells on a 96-well plate (3 ×10^4^ cells/cm^2^) were acquired automatically with an Operetta® High Content Imaging System (PerkinElmer, UK). After 2 days of culture, cells were treated with free DOX (15 μg/ml) or LIP formulations (DOX 15 μg/ml), and the uptake was evaluated by acquiring images at three different time points (0, 1, and 3 h). Before imaging, cells were washed three times with PBS. Images were acquired in the DOX channel and in the brightfield channel, using a 40× air objective lens and standard instrument filters. Ten different fields were imaged in each well. The image were then analyzed by the Harmony® analysis software (PerkinElmer, UK).

### Actin, Tubulin, and DAPI Staining

NHA and U87-MG cells were plated at a density of 5 ×10^3^ or 1.5 ×10^4^ cells/cm^2^, respectively, on porcine gelatin pretreated coverslips. One day after seeding, cells were fixed for 10 min with 3.7% paraformaldehyde in PBS, permeabilized for 4 min with 0.1% Triton X-100/PBS, and finally stained with different antibodies. In particular, cells were treated with TRITC-phalloidin (1:40 in 1% BSA/PBS) for actin staining, as described previously (Ceriani et al., 2007). For tubulin staining, cells were incubated with mouse monoclonal anti-β-tubulin primary antibody (1:150 in 1% BSA/PBS) for 1 h at 37°C. Then cells were washed and incubated with the secondary antibody Alexa Fluor 488 goat anti-mouse IgG (1:200 in 1% BSA/PBS) for 45 min at 37°C.

For nuclei staining, U87-MG and NHA cells were plated on gelatin pretreated coverslips. Cells were leaved in culture complete medium for 48 h and then incubated with 15 μg/ml of free DOX for 1 h. Cells were then stained for 20 min with DiO (5 μl/ml), fixed, permeabilized, and colored with DAPI (Sigma) (1 μg/ml) for 10 min at room temperature.

### Fluorescence-Activated Cell Sorting Analysis of Cell-to-Cell Liposome Transfer

U87-MG cells and NHA cells were seeded on 12-well plates at a cell density of 6.5 ×10^3^ and 15 ×10^4^ cells/cm^2^, respectively. Three days after seeding, “donor” cells were incubated with BODIPY-Sm Mf-LIP (200 nmol of total lipids) for 1 h at 37°C, whereas “acceptor” cells were treated with CellTrace Far Red Dye (CT) at 1 μM for NHA and 10 μM for U87-MG cells for 30 min. Cells were detached, and the following co-culture between “donor” and “acceptor” cells was set up: U87-MG (donor) → NHA (acceptor); U87-MG (donor) → U87-MG (acceptor); and NHA (donor) → NHA (acceptor). Co-culture were maintained for 24 h at 37°C. Cell-to-cell transfer of Mf-LIP was assessed by fluorescence-activated cell sorting (FACS) (FACSCantoI BD Biosciences) analysis. At least 50,000 events were acquired in an operator-defined gate designed on a physical parameter (forward versus side scatter [FSC vs. SSC]) dot plot. The fluorescence intensity analysis on fluorescein isothiocyanate (FITC) (to detect BODIPY) and allophycocyanin (APC) (to detect CT) histogram was performed on a single cell gate defined on a FSC-H vs. FSC-A dot plot. The reported data refer only to the double FITC/APC-positive events among this population, representing the Mf-LIP transfer to “acceptor” cells.

### Statistical Analysis

For TnT quantification, data were analyzed by Student *t*-test. Data were expressed as a mean ± standard error (SE). For percentage distribution of thin and thick TnTs, data were analyzed by two-way or one-way ANOVA followed respectively by Sidak's multiple comparisons test or Dunnett's *post-hoc* test.

All experiments were conducted at least in triplicate. All the analyses were performed with GraphPad Prism 8 software (license number: GP8-1519368-RFQS-B8CB4). Differences were considered significant at ^*^p < 0.05, ^**^p < 0.01, and ^***^p < 0.001.

## Results

### Characterization of Liposomes

The results showed that DOX-LIP displayed a diameter of 121 ± 6 nm with a PDI value of 0.098 ± 0.01; the ζ-potential was −19.32 ± 0.58 mV. Mf-LIP showed a diameter of 187 ± 5 nm with a PDI value of 0.087 ± 0.05; the ζ-potential was −14.5 ± 0.43 mV. These parameters remained constant for 1 week within the experimental error (<2.7% of variation). For both preparations, the total lipid recovery after purification was 79.5 ± 8%. For Mf-LIP, the yield of functionalization with mApoE and ClTx was 88.5 ± 10% (corresponding to 2.2 mol% of mApoE/total lipids) and 71.2 ± 3% (corresponding to 1.42 mol% of ClTx/total lipids), respectively. For DOX-LIP, the incorporation yield of DOX was 70 ± 6%, corresponding to 80 ± 5 μg of DOX/μmol of lipids. These results derived from at least five different batches.

### U87-MG Cells, Compared With Normal Human Astrocytes, Form Tunneling Nanotubes With Different Thickness

**Figure 1:**
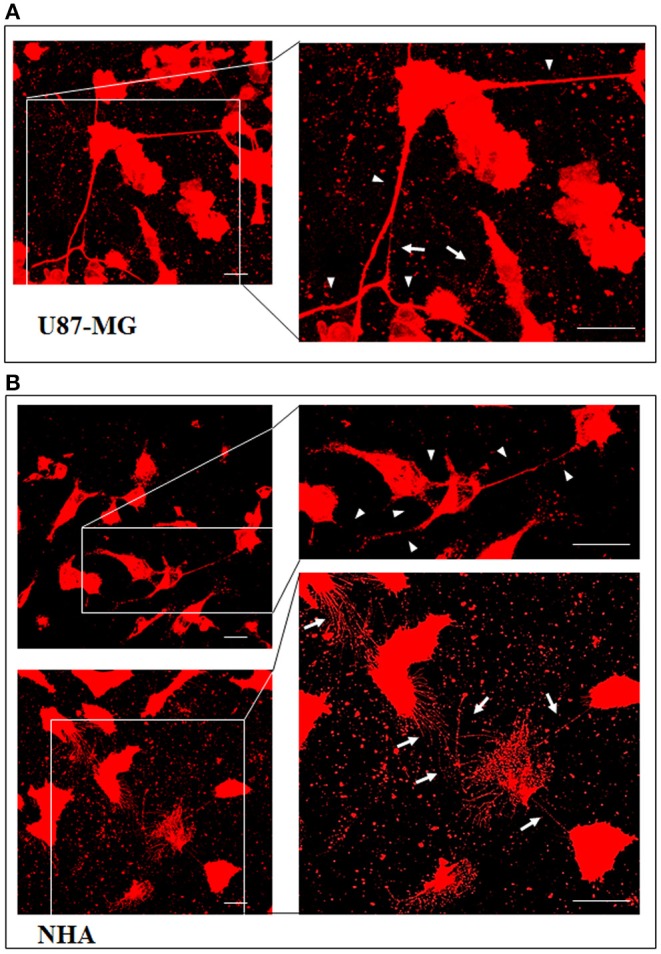
U87-MG and normal human astrocyte (NHA) cells form thin and thick tunneling nanotubes (TnTs). U87-MG cells **(A)** or NHA cells **(B)** were plated on gelatin pretreated coverslips and then fixed and stained with 1,1′-dioctadecyl-3,3,3′ ,3′-tetramethylindocarbocyanine perchlorate (DiI) (1.9 μl/ml) to detect TnTs. Fluorescence images were acquired by a 40× magnification on A1R Nikon laser scanning confocal microscope. The images show the maximum projection obtained from the z-projections shown in Figures S1, S2. White arrows indicate thin TnTs, whereas white triangles indicate thick TnTs. Scale bar: 10 μm. Magnified views of protrusions are shown.

**Figure 2:**
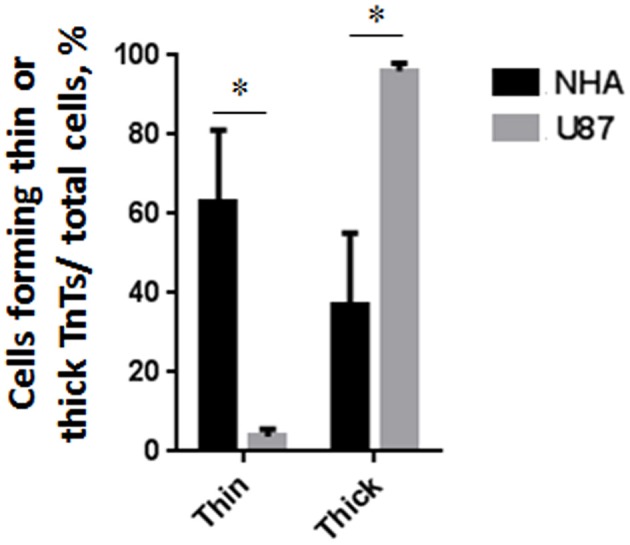
Percentage distribution of thin and thick tunneling nanotubes (TnTs) in U87-MG and normal human astrocyte (NHA) cells. Percentage of U87-MG cells and NHA cells forming thin or thick TnTs on total cells is shown. At least 200 cells were analyzed per group in three independent experiments. Data are expressed as mean ± SE from three independent experiments. Data were analyzed by two-way ANOVA followed by Sidak's multiple comparisons test; *p < 0.05.

To investigate if U87-MG cells (model of GBM tumor cells) are able to form *in vitro* intercellular connections with characteristics of TnTs, and if they are different from those formed by NHA cells (model of normal healthy astrocytes), we used confocal microscopy technique and 3D reconstruction. Both cell types form protrusions connecting distant cells with characteristics of TnTs (Figure 1), which were not in contact with the substratum (Figures S1, S2). To allow for a quantitative determination, the observed membrane protrusions of about 200 cells were scored for each cell line. The results showed that the number of cells forming TnTs is comparable between U87-MG and NHA (44 ± 6.6 and 57 ± 3.5%, respectively) (Figure S3). Confocal images show the presence of TnTs of different thickness, very thin (≤0.7 μm, measuring a minimum of 100–200 nm) and thick (≥0.7 μm, up to 1 μm) (Gerdes et al., 2007). More interestingly, we detected significant differences in both thin and thick TnTs: U87-MG cells formed almost exclusively thick protrusions, whereas NHA formed either thin and thick TnTs (Figure 2). The measurement of TnT diameter by light microscopy was not accurate owing to the resolution limit. Confocal microscopy showed that some TnTs reach thicknesses of over 700 nm, which could be due to incorporation of additional components inside the TnTs, such as microtubules, as previously suggested (Önfelt et al., 2006).

To evaluate the presence of tubulin, typical marker for thick TnTs, and of actin, typical marker for thin TnTs (D'Aloia et al., 2018), U87-GM and NHA cells were stained with anti-tubulin fluorescent antibody and fluorescent phalloidin.

**Figure 3:**
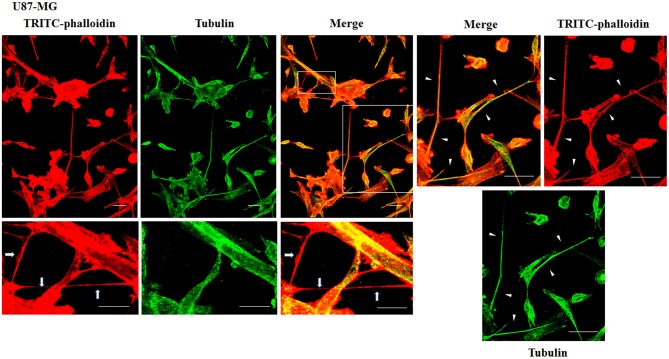
Characterization of tunneling nanotubes (TnTs) in U87-MG cells. U87-MG cells were plated on gelatin pretreated coverslips. Cells were fixed, permeabilized, and immunostained with either the anti-β-tubulin antibody (1:150) or TRITC-phalloidin (1:40) to detect microtubules and actin filaments. Fluorescence images were captured by confocal microscopy. White triangles indicate thick TnTs, and white arrows indicate thin TnTs. Magnified views of protrusions are shown for each channel. Scale bar: 10 μm.

**Figure 4:**
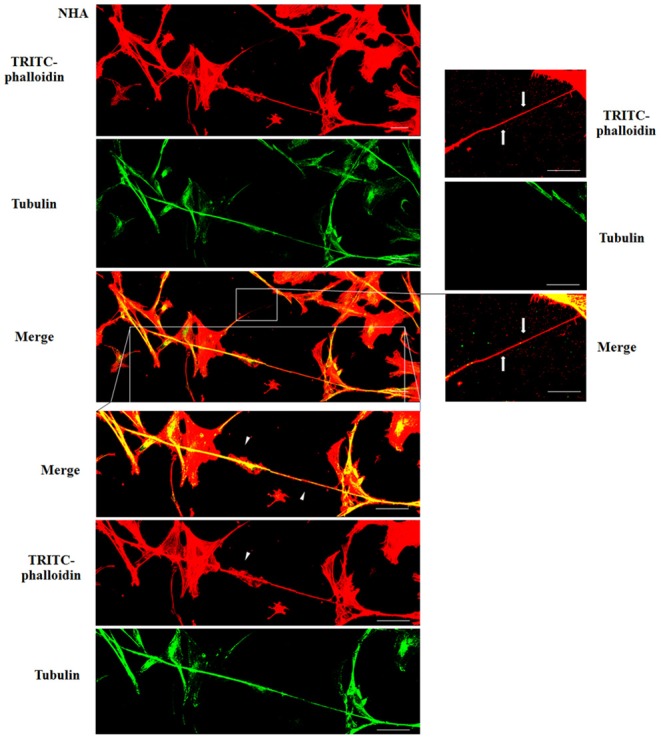
Characterization of tunneling nanotubes (TnTs) in normal human astrocyte (NHA) cells. NHA cells were plated on gelatin pretreated coverslips. Cells were fixed, permeabilized, and immunostained with either the anti-β-tubulin antibody (1:150) or TRITC-phalloidin (1:40) to detect microtubules and actin filaments. Fluorescence images were captured by confocal microscopy. White triangles indicate thick TnTs, and white arrows indicate thin TnTs. Magnified views of protrusions are shown for each channel. Scale bar: 10 μm.

The results showed that U87-MG cells were able to form thick TnTs, which contained both actin and tubulin (Figure 3). NHA cells were able to form thick TnTs made of actin and tubulin, but they also established thin TnTs, which were positive only to actin staining (Figure 4).

### Doxorubicin Treatment Induced Changes in the Tunneling Nanotube Thickness of U87-MG Cells

**Figure 5:**
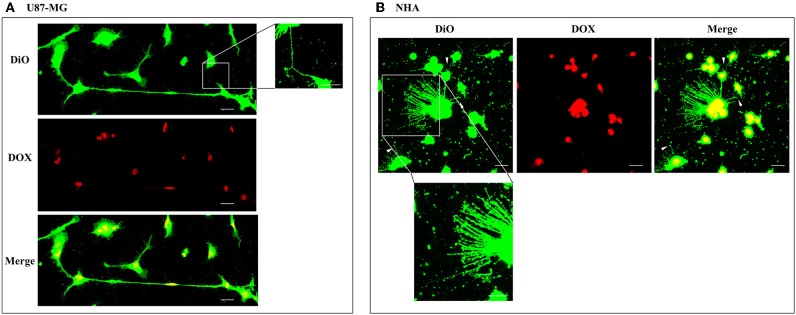
Doxorubicin localizes into the nucleus of U87-MG and normal human astrocyte (NHA) cells. U87-MG cells **(A)** and NHA cells **(B)** were plated on gelatin pretreated coverslips, incubated with 15 μg/ml of DOX for 1 h, and then stained with 3,3′-dioctadecyloxacarbocyanine perchlorate (DiO) (5 μl/ml) to detect tunneling nanotubes (TnTs). Cells were fixed, and fluorescence images were captured with a 40× magnification on A1R Nikon laser scanning confocal microscope. White triangles indicate thin TnTs. Magnified views of thin TnT protrusions are shown. Scale bar: 10 μm. DOX = doxorubicin.

**Figure 6:**
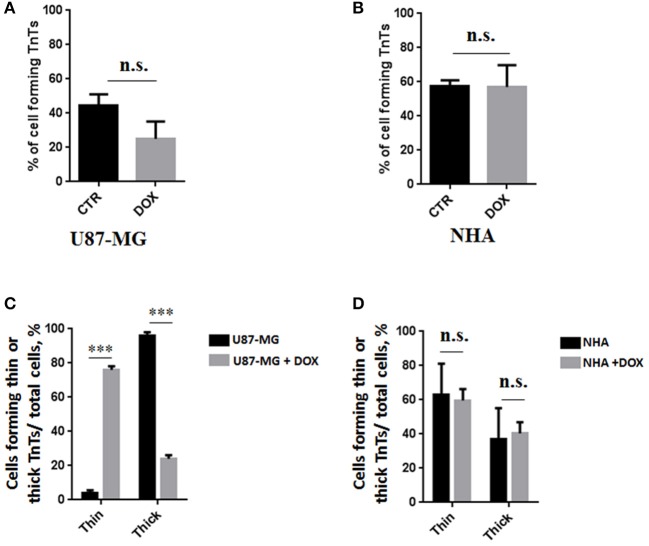
Percentage of cells forming tunneling nanotubes (TnTs) after DOX treatment. Percentage of U87-MG cells **(A)** and normal human astrocytes (NHA) cells **(B)** forming TnTs on total cells is shown. At least 200 cells were analyzed per group in three independent experiments. Data are expressed as mean ± SE from three independent experiments. Data were analyzed by Student *t*-test; n.s., not significant. DOX, doxorubicin; CTR, control untreated cells. **(C,D)** Percentage distribution of thin and thick TnTs in U87-MG **(C)** and NHA **(D)** cells on total cells, after treatment with DOX. At least 200 cells were analyzed per group in three independent experiments. Data are expressed as mean ± SE from three independent experiments. Data were analyzed by two-way ANOVA followed by Sidak's multiple comparisons test; n.s., not significant; ***p < 0.001.

To evaluate the ability of U87-MG and NHA to exchange DOX via TnTs, cells were treated with two different doses of free DOX. Treatments with 25 μg/ml of DOX for 24 h induced a strong toxic effect on both cell types, hindering the image analysis (data not shown). Then, all the subsequent experiments were carried out by incubating cells with 15 μg/ml of DOX for 1 h. Analysis performed at confocal microscope showed that DOX (λex = 495 nm; λem = 592 nm) localizes principally at the nucleus in both cell lines (Figure 5; Figure S4), as expected (de Lange et al., 1992), but it was not detectable along TnT structures. The quantitative determination of TnTs revealed that the % of cells forming TnTs was not affected by the treatment with free DOX (Figures 6A,B), for both the cell types used.

Comparing the thickness of TnTs, the DOX treatment on U87-MG cells induced the formation of about 80% of thin TnTs, with a strong reduction of thick TnTs (Figure 6C). By prolonging the incubation time up to 24 h, TnTs disappeared and U87-GM cells died (Figure S5).

No significant changes in TnTs thickness were detected for NHA, which remained comparable with untreated NHA (Figure 6D).

### Multifunctional Liposomes, Compared With Normal Human Astrocytes, Were Preferentially Located in the Thickest Tunneling Nanotubes of U87-MG

**Figure 7:**
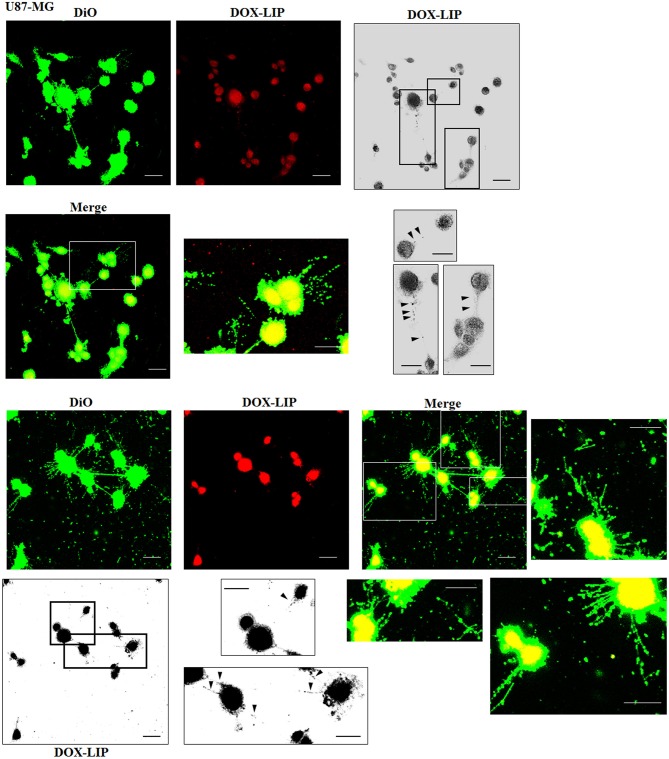
Localization of DOX-LIP in U87-MG tunneling nanotubes (TnTs). U87-MG cells were plated on gelatin pretreated coverslips. Cells were left in culture complete medium for 48 h and then incubated with DOX-LIP (15 μg/ml of DOX and 200 nmol of total lipids) for 1 h. Cells were later stained for 20 min with 3,3′-dioctadecyloxacarbocyanine perchlorate (DiO) (5 μl/ml) and fixed, and fluorescence images were captured with a 40× magnification on A1R Nikon laser scanning confocal microscope. Black triangles indicate the DOX-LIP in thick TnTs. Magnified views of protrusions and black-and-white images are shown. Scale bar: 10 μm. DOX-LIP, liposomes embedding doxorubicin.

**Figure 8:**
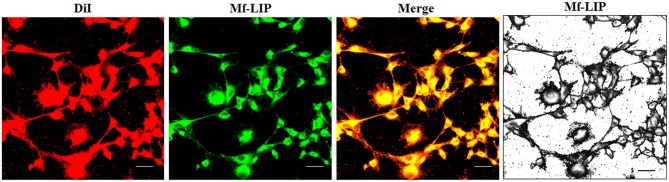
Localization of Mf-LIP in U87-MG tunneling nanotubes (TnTs). U87-MG cells were plated on gelatin pretreated coverslips. Cells were left in culture complete medium for 48 h, and then cells were incubated with Mf-LIP (200 nmol of total lipids) for 1 h. Cells were later stained for 20 min with 1,1′-dioctadecyl-3,3,3′ ,3′-tetramethylindocarbocyanine perchlorate (DiI) (1.9 μl/ml). Cells were fixed, and fluorescence images were captured with a 40× magnification on A1R Nikon laser scanning confocal microscope. Black-and-white image is also shown. Scale bar: 10 μm. Mf-LIP, multifunctional liposomes.

**Figure 9:**
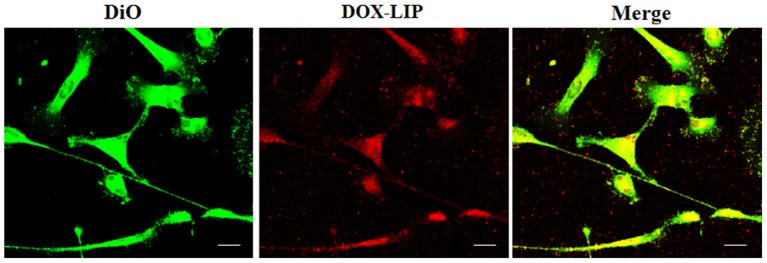
Localization of DOX-LIP in normal human astrocytes (NHA) tunneling nanotubes (TnTs). NHA cells were seeded on gelatin pretreated coverslips. Cells were left in culture complete medium for 48 h and then incubated with DOX-LIP (15 μg/ml of DOX and 200 nmol of total lipids) for 1 h. Cells were later stained for 20 min with 3,3′-dioctadecyloxacarbocyanine perchlorate (DiO) (5 μl/ml) and fixed, and fluorescence images were captured with a 40× magnification on A1R Nikon laser scanning confocal microscope. Scale bar: 10 μm. DOX-LIP, liposomes embedding doxorubicin.

**Figure 10:**
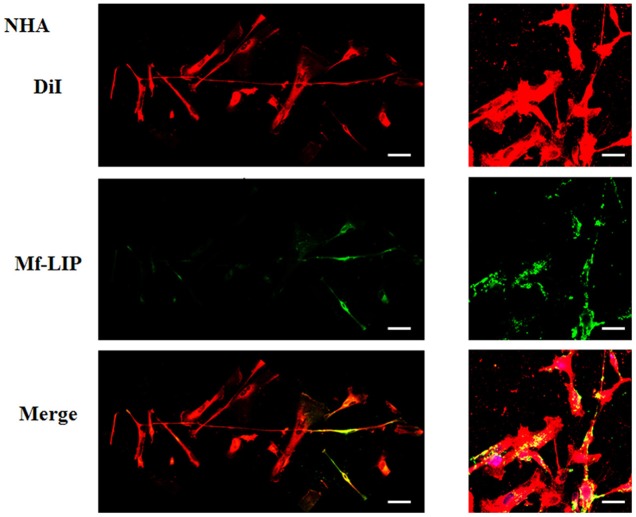
Localization of Mf-LIP in normal human astrocytes (NHA) tunneling nanotubes (TnTs). NHA cells were plated on gelatin pretreated coverslips. Cells were left in culture complete medium for 48 h and then incubated with Mf-LIP (200 nmol of total lipids) for 1 h. Cells were later stained for 20 min with 1,1′-dioctadecyl-3,3,3′ ,3′-tetramethylindocarbocyanine perchlorate (DiI) (1.9 μl/ml). Cells were fixed, and fluorescence images were captured with a 40× magnification on A1R Nikon laser scanning confocal microscope. Black triangles indicate the presence of Mf-LIP in thick TnTs. Magnified views of protrusions and black-and-white images are shown. Scale bar: 10 μm. Mf-LIP, multifunctional liposomes.

**Figure 11:**
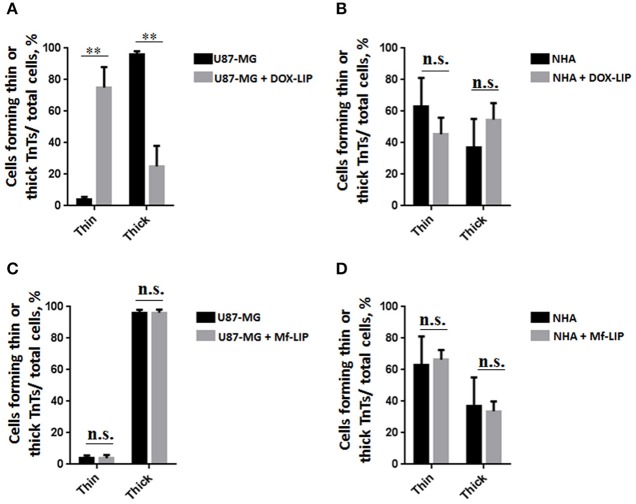
Percentage distribution of thin and thick tunneling nanotubes (TnTs) in U87-MG and normal human astrocytes (NHA) cells after treatment with liposomes embedding doxorubicin (DOX-LIP) or multifunctional liposomes (Mf-LIP). Percentage distribution of thin and thick TnTs in U87-MG **(A)** and NHA **(B)** cells after treatment with DOX-LIP. Percentage distribution of thin and thick TnTs in U87-MG **(C)** and NHA **(D)** cells after treatment with Mf-LIP. At least 200 cells were analyzed per group in three independent experiments. Data are expressed as mean ± SE from three independent experiments. Data were analyzed by two-way ANOVA followed by Sidak's multiple comparisons test; n.s., not significant; **p < 0.01.

The cellular uptake of Mf-LIP (LIP bi-functionalized with mApoE and ClTX) by U87-MG was evaluated by confocal microscopy and fluorescence measurements. The results showed that Mf-LIP displayed a three-fold increase of cellular uptake, compared with DOX-LIP used as a control (Figure S6). Both DOX-LIP and Mf-LIP were localized only in thickest TnTs (Figures 7, 8). Contrarily, NHA cells were able to uptake only a small amount of DOX-LIP and Mf-LIP. Also in these cells, LIP were localized only in thick TnTs (Figures 9, 10). The treatment of U87-MG cells with DOX-LIP did not affect the % of cells forming TnTs (Figure S7A) but strongly increased thin TnTs, with a significant reduction of thick TnTs (Figures 11A,B), similar to the effect exerted by free DOX.

**Figure 12:**
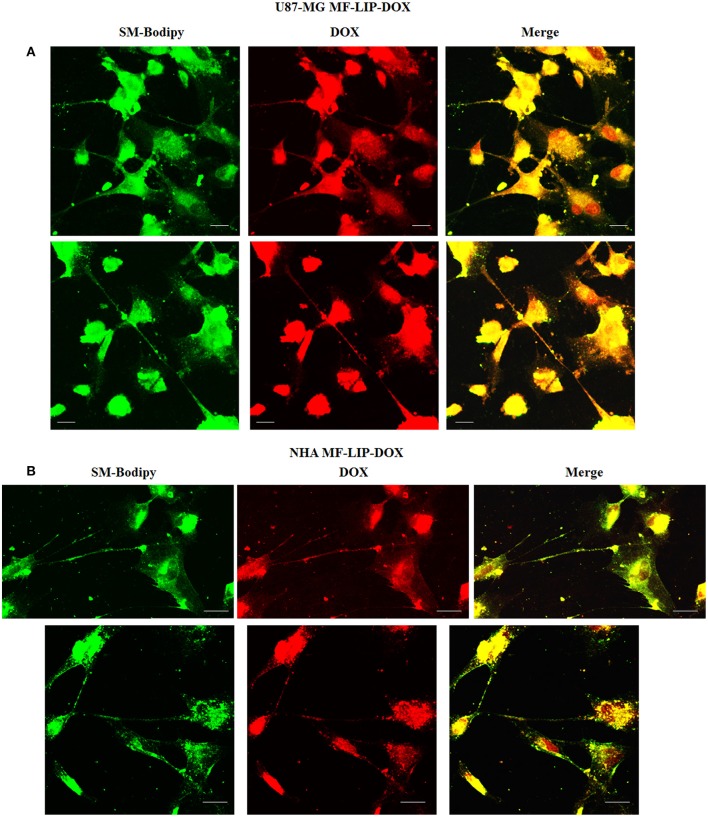
Multifunctional liposomes (Mf-LIP) embedding doxorubicin (DOX) localized in tunneling nanotubes (TnTs) formed by U87-MG and normal human astrocytes (NHA) cells. U87-MG **(A)** and NHA **(B)** cells were plated on gelatin pretreated coverslips. Cells were left in culture complete medium for 48 h and then incubated with Mf-LIP embedding DOX (15 μg/ml of DOX and 200 nmol of total lipids) for 1 h. Cells were fixed and fluorescence images were captured with a 40× magnification on A1R Nikon laser scanning confocal microscope. Scale bar: 10 μm. BODIPY-Sm = sphingomyelin present in Mf-LIP conjugate with fluorophore BODIPY.

The treatment of U87-MG and NHA cells, compared with untreated cells, with Mf-LIP did not change the percentage of thin and thick TnTs (Figures 11C,D). Also the treatment with Mf-LIP did not affect the % of cells forming TnTs (Figure S7A) neither the ratio between thin and thick TnTs (Figures 11C,D). No differences were detected in TnTs formed by NHA cells after incubation with DOX-LIP or Mf-LIP (Figure S7B; Figures 6, 11C,D). Finally, to evaluate the integrity of LIP inside to TnTs, double-labeled Mf-LIP (containing BODIPY-Sm and DOX) were used. As it is shown in Figure 12, the co-localization of both fluorescent signals was detected in TnTs from both U87-MG and NHA cells.

### Multifunctional Liposomes Were Preferentially Exchanged via Tunneling Nanotubes Between U87-MG Co-cultured Cells

**Figure 13:**
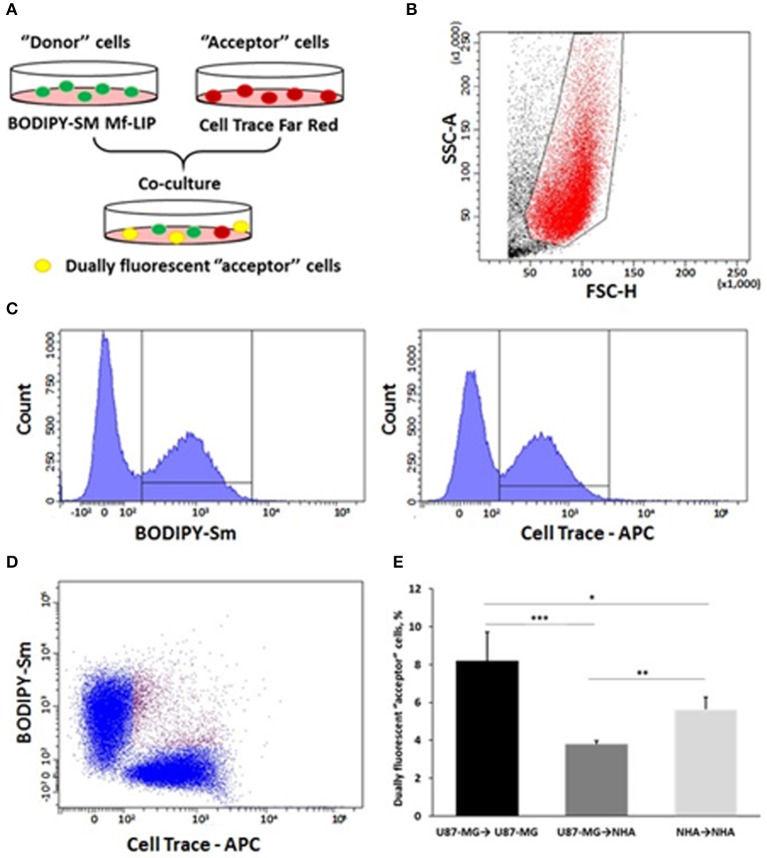
Fluorescence-activated cell sorting (FACS) analysis of multifunctional liposomes (Mf-LIP) exchange in co-culture systems. **(A)** representative image of the experimental set-up: “donor” cells were incubated with BODIPY-Sm Mf-LIP (200 nmol total lipids) for 1 h at 37°C, whereas “acceptor” cells were treated with CellTrace Far Red Dye (CT) at 1 μM for normal human astrocytes (NHA) and 10 μM for U87-MG cells for 30 min. Cells were detached, and the following co-culture between “donor” and “acceptor” cells was set up: U87-MG (donor) → NHA (acceptor); U87-MG (donor) → U87-MG (acceptor); NHA (donor) → NHA (acceptor); and NHA (donor) → U87-MG (acceptor). Cell-to-cell transfer of Mf-LIP was assessed by FACS analysis. **(B)** The events to be analyzed (red dots) were selected on a physical parameter dot plot using an operator-defined gate. A further analysis was performed on a FSC-A vs. FSC-H dot plot to focus on the signal derived from single cells (not included). **(C)** The gated events were analyzed for fluorescence intensity on a fluorescein isothiocyanate (FITC) (BODIPY) and an allophycocyanin (APC) (Cell Trace) channel. **(D)** For each fluorescence, a single positive events' population was selected on the corresponding histograms, and the intersection highlights the double-positive events on a BODIPY vs. Cell Trace dot plot (purple dots). **(E)** Percentage of dually fluorescent “acceptor” cells that received Mf-LIP from the corresponding “donor” population. *N* = 3 independent experiments. *p < 0.05; **p < 0.01; ***p < 0.001 by Student *t*-test.

In order to prove the ability of cells to mutually exchange Mf-LIP, co-culture mixtures between U87-MG and NHA were set up. U87-MG or NHA cells were incubated with fluorescent-labeled Mf-LIP in order to generate Mf-LIP-loaded “donor” populations. U87-MG or NHA cells were labeled with a fluorescent cell dye to generate detectable “acceptor” populations. Then, different co-cultures of “donor” and “acceptor” cells were prepared (Figure 13A). The cell-to-cell transfer of Mf-LIP was assessed by FACS analysis to detect the co-presence of the two fluorescent signals in the “acceptor” cells (Figures 13B–D).

The results showed that Mf-LIP were transferred via TnTs between NHA (donor) → NHA (acceptor) with a low rate, as demonstrated by the detection of 5 ± 0.6% of double fluorescent cells over the total cells. In comparison, the rate of Mf-LIP exchange of U87-MG (donor) → NHA (acceptor) was lower (3.8 ± 0.2% of Mf-LIP positive “acceptor” NHA).

Interestingly, the results showed that U87-MG cells were more efficient in transferring Mf-LIP between them than were U87-MG (donor) → NHA (acceptor), as demonstrated by the detection of 8.2 ± 1.5% of Mf-LIP positive “acceptor” U87-MG (Figure 13E).

## Discussion

In the context of searching more effective therapies against GBM, which remains an incurable brain tumor, we focus our attention on the cell communication. Intercellular communication plays an important role in tumor progression, invasiveness, and resistance to conventional treatments (Broekman et al., 2018). Among the different ways that cells used to exchange non-secretable messages, TnTs and TmTs are involved in the re-growth of GBM after surgery and in conferring resistance to radiotherapy and chemotherapy (Moschoi et al., 2016; Weil et al., 2017). Starting from our expertise in the design of nanoparticles, we synthesized and characterized LIP carry doxorubicin, as an anti-cancer drug model, and dually functionalized with mApoE and with ClTx, as GBM targeting ligands (DeBin et al., 1993; Maletínská et al., 2000; Lyons et al., 2002; Ojeda et al., 2016; Formicola et al., 2019). The ability of human primary glioblastoma cell line (U87-MG), in comparison with NHA, to exchange Mf-LIP via TnTs has been investigated. Mf-LIP characterization has shown that the different batches herein prepared were highly reproducible and stable over time, with the yield of the reactions comparable with those of previously reported ones (Re et al., 2010, 2011; Formicola et al., 2019).

As it is reported in literature that TnTs are not observed in some glioma cellular models (Van der Vos et al., 2016), we checked if U87-MG cells and NHA cells herein used were able to form TnTs *in vitro*. The results showed that both types of cells were able to communicate between them by TnTs, as already reported (Zhang and Zhang, 2015; Rostami et al., 2017; Reindl et al., 2019), and the percentage of cells forming TnTs was similar between U87-MG and NHA cells.

As TnTs have been grouped into two main classes, very thin (≤0.7 μm, measuring a minimum of 100–200 nm) and thick (≥0.7 μm, up to 1 μm; Gerdes et al., 2007), we analyzed the heterogeneity of TnTs formed by U87-MG and NHA.

Structural analysis and the comparison of the thickness of TnTs formed by these cells have shown that U87-MG cells formed almost exclusively thick protrusions, whereas NHA formed either thin or thick TnTs.

Considering that thick TnTs are more efficient in transport of molecules and organelles (Veranic et al., 2008; Mittal et al., 2019), this difference could may be exploited to increase the range of drug delivery between cancer cells. Moreover, TnTs are also classified according to their different morphology/function in TnT type I, short dynamic structures, containing actin filament and engaged in exploring the surrounding microenvironment, and TnT type II, which are longer and more stable processes, containing actin and tubulin filaments and apparently involved in organelles shuttle (Veranic et al., 2008). Here, reported immunofluorescence experiments staining actin and tubulin showed that U87-MG mainly formed TnT type II, compared with NHA, which formed mostly TnT type I. Accordingly, U87-MG cells were able to better exchange Mf-LIP, as shown by the detection of LIP-associated fluorescence in thick TnTs. Moreover, we showed that the LIP surface functionalization with mApoE and ClTx strongly increased the cell uptake by U87-MG, whereas no differences were detected with NHA in terms of LIP uptake. This suggest that the presence of these two ligands may promote a more specific targeting of cancer cells, probably owing to the overexpression of lipoprotein (LDL)-receptor by U87-MG cells (DeBin et al., 1993; Maletínská et al., 2000), which is the target ligand of mApoE peptide, and ClTx, which has been shown to selectively bind a specific chloride channel on glioma cell surface (Lyons et al., 2002; Xiang et al., 2011; Ojeda et al., 2016).

Moreover, the encapsulation of DOX in LIP facilitates its passage through TnTs, with respect to free DOX, which remains almost exclusively localized in the nuclear region. Considering that cells physiologically produced TnTs under stress conditions (e.g., hypoxia conditions, drugs, and oxidative stress), we assessed the effect of DOX treatment on TnTs formed by U87-MG cells. The results showed that free DOX and DOX-LIP induced the formation of thin TnTs, with a strong reduction of thick TnTs, and by prolonging the incubation time, TnTs disappeared and U87-MG cells died as also shown for other cell types (Rustom, 2016). By comparing these results with those obtained in NHA cells, Mf-LIP were localized in TnTs with a little extent, and the few LIP inside in NHA's TnTs were again localized in the thick ones. This corroborates the fact that thick TnTs are mainly involved in the intercellular trafficking of drug-loaded LIP.

More appealing is that the structural difference between TnTs formed by GBM cells and NHA could be useful to design precise and specific nanotherapeutics.

As a proof-of-concept, the ability of cells to mutually exchange Mf-LIP was evaluated in different co-culture mixtures. Interestingly, the results showed that U87-MG cells were more efficient in transferring Mf-LIP between them, compared with the Mf-LIP exchange between healthy astrocytes. More excitingly, the transport of Mf-LIP via TnTs preferentially occurred from U87-MG to U87-MG, than toward NHA. This opens the possibility to exploit TnTs as drug-delivery channels, thus improving the cancer therapy. In particular, this strategy can be useful to reach isolated, infiltrating tumor cells that are hardly targeted by drug diffusion in the brain parenchyma. Nowadays, few papers are available showing the involvement of TnTs-mediated intercellular transport of nanoparticles (Kristl et al., 2013; Epperla et al., 2015; Deng et al., 2018), and none of them is dedicated to the comparison between healthy and tumor cells in nanoparticles trafficking. It is important to highlight that all the results herein reported were obtained using one GBM-derived cell line, which are not fully representative of human GBM. For this reason, the validation of these results will be further performed on patient-derived glioblastoma cells, with stem cells included. Moreover, even with the detected TnTs connecting distant cells without being in contact with the substratum (as shown by z-stack imaging), the same analysis conducted in a 3D context could be beneficial to deeply investigate this issue.

In conclusion, the understanding of the possible intercellular delivery of nanotherapeutics cargo via TnTs can significantly influence the approaches to treat specific diseases.

## Data Availability Statement

All datasets generated for this study are included in the article/Supplementary Material.

## Author Contributions

BF performed the preparation and characterization of all liposomal formulations used. AD'A performed confocal microscopy experiments to detect TnTs. RD set up the cell culture conditions. SS performed statistical analysis of data. RR performed FACS analysis. MC and FR contributed to the data interpretation and participated in the drafting of the manuscript. FR coordinated the study, designed the experiments, and analyzed the data. All authors contributed to the paper revision, read and approved the submitted version, and agreed to be accountable for all the aspects of the work.

### Conflict of Interest

The authors declare that the research was conducted in the absence of any commercial or financial relationships that could be construed as a potential conflict of interest.
