# Supplementary material for: Differential Exchange of Multifunctional Liposomes Between Glioblastoma Cells and Healthy Astrocytes via Tunneling Nanotubes
Source: Front Bioeng Biotechnol. 2019 Dec 12;7:403. doi: 10.3389/fbioe.2019.00403 (PMC6920177; doi:10.3389/fbioe.2019.00403)
Supplement: Supplementary file 1 [file Data_Sheet_1.pdf]

Supplementary Material

1. Supplementary Data and Figures

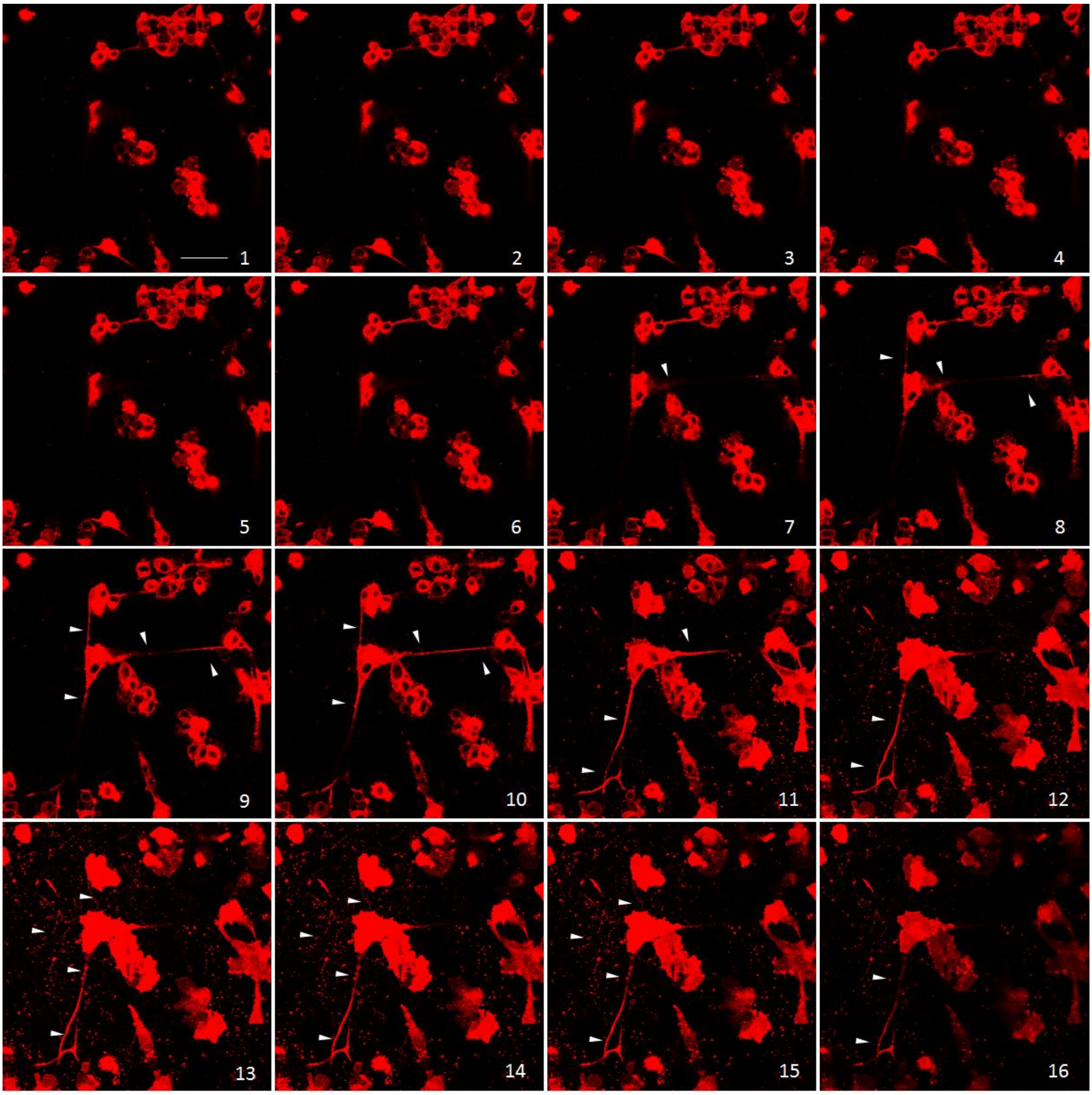

Figure S1. Series of confocal z-slides. U87-MG cells were plated on gelatin pre-treated coverslips. Cells were stained with the membrane dye DiI, fixed, and imaged via confocal microscopy. Series of DiI-stained confocal z-slides and maximum intensity projection of z-slides from U87-MG cells. White triangles indicate TnTs. Scale bar: 10 µm. The step size is 0.625 µm and the number of optical section is 16.

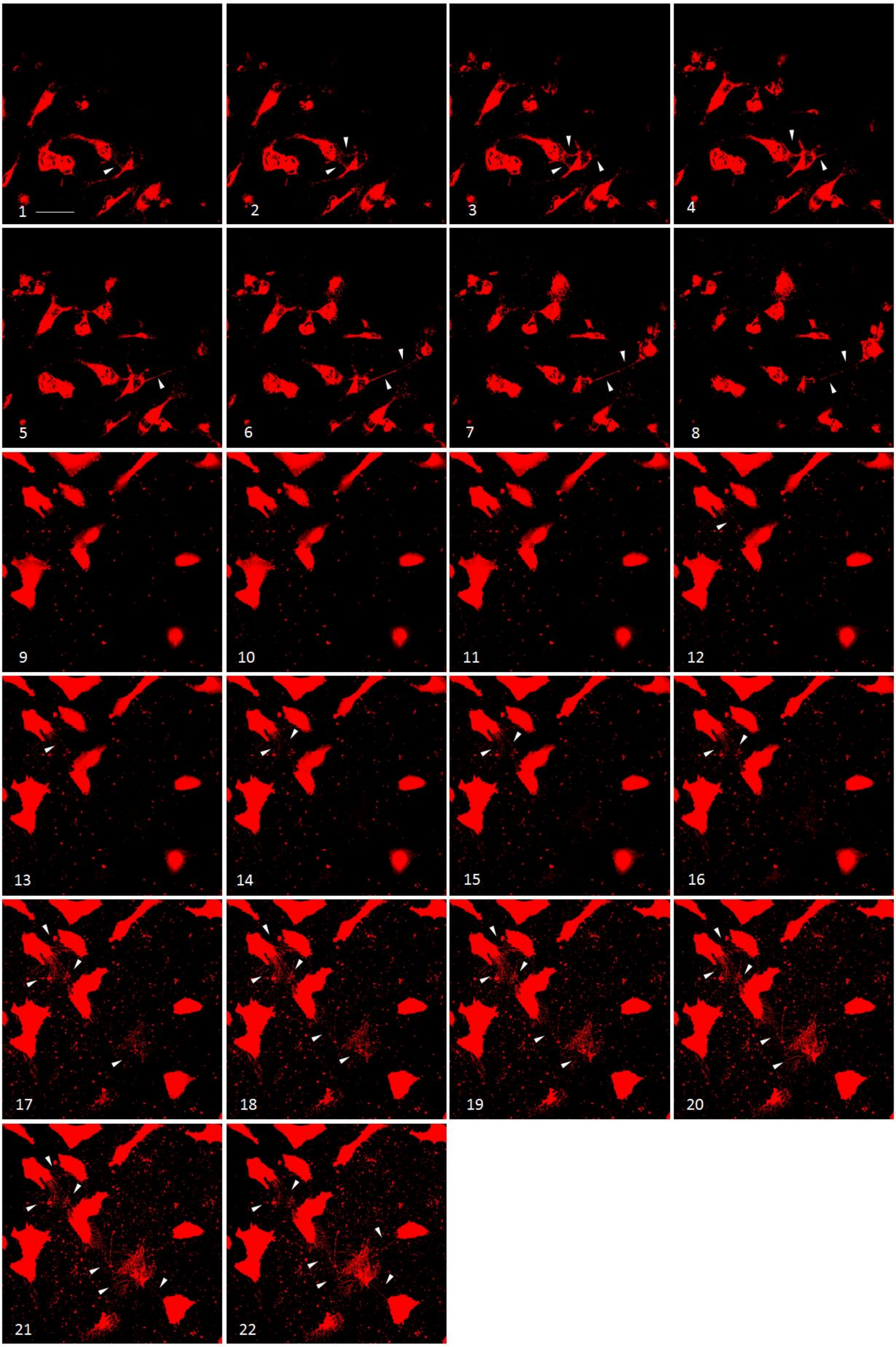

1
2
3
4
5
6
7
8
9
10
11
12
13
14
15
16
17
18
19
20
21
22

Figure S2. Series of confocal z-slides. NHA cells were plated on gelatin pre-treated coverslips. Cells were stained with the membrane dye DiI, fixed, and imaged via confocal microscopy. Series of DiI-stained confocal z-slides and maximum intensity projection of z-slides from NHA cells. White triangles indicate TnTs. Scale bar: 10 μm. The step size is 0.454 μm and the number of optical section is 22.

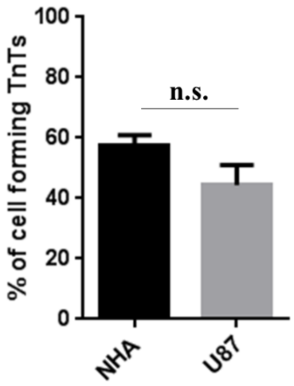

Figure S3. Percentage of cells forming TnTs. Percentage of U87-MG cells and NHA cells forming TnTs on total cells is shown. At least 200 cells were analyzed per group in three independent experiments. Data are expressed as mean ± S.E from three independent experiments. Data were analyzed by Student *t* test; n.s.= not significant.

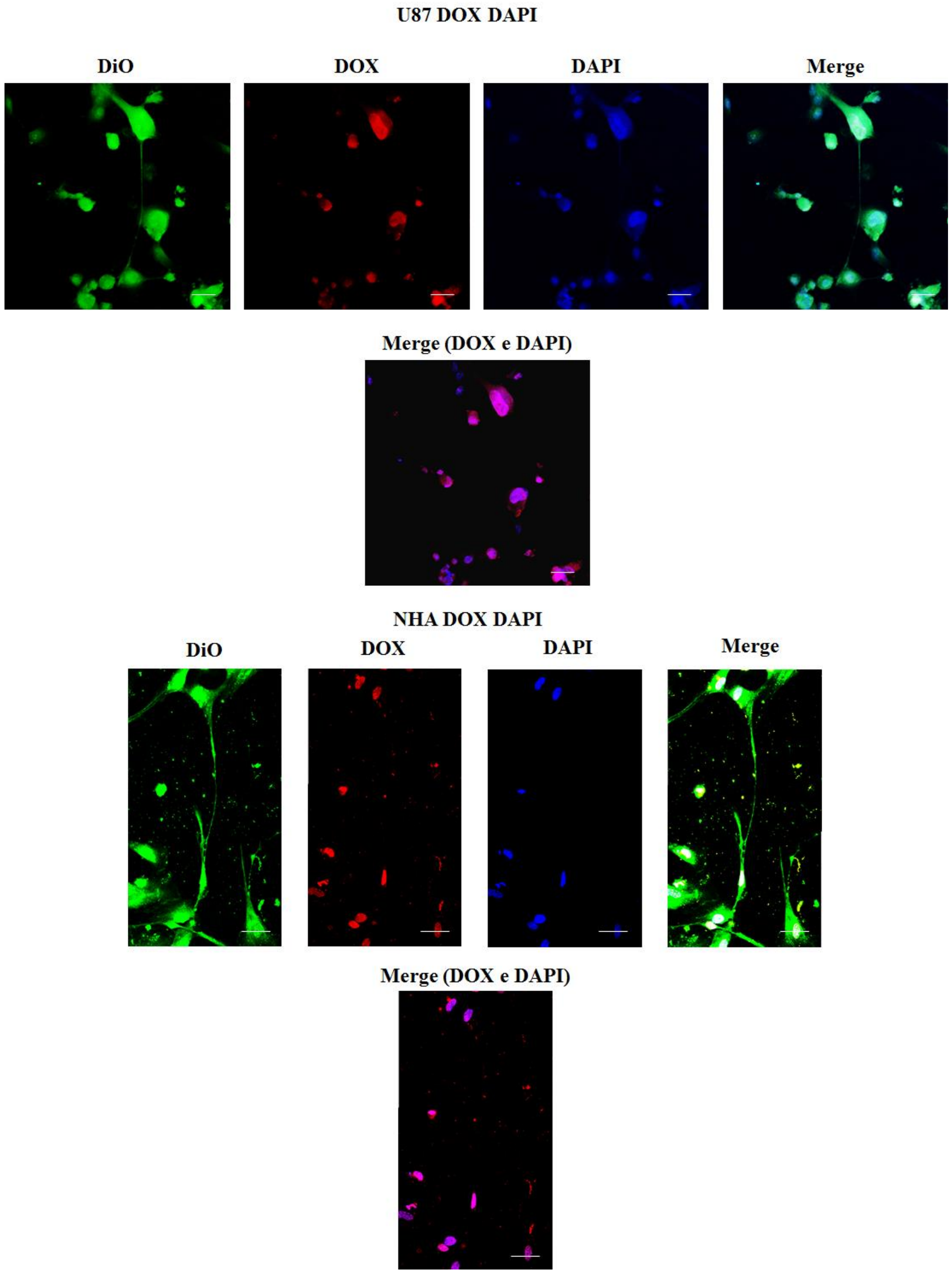

Figure S4. Doxorubicin (DOX) localizes principally at the nucleus in U87-MG and NHA cell lines. U87-MG and NHA cells were plated on gelatin pre-treated coverslips. Cells were leaved in culture complete medium for 48 h and then incubated with 15 µg/ml of free DOX for 1 h. Cells were later stained for 20 min with DiO (5 µl/ml), fixed, permeabilized and colored with DAPI (1µg/ml) for 10 minutes. Fluorescence images were captured with a 40x magnification on A1R Nikon laser scanning confocal microscope. Scale bar: 10µm. DOX = doxorubicin.

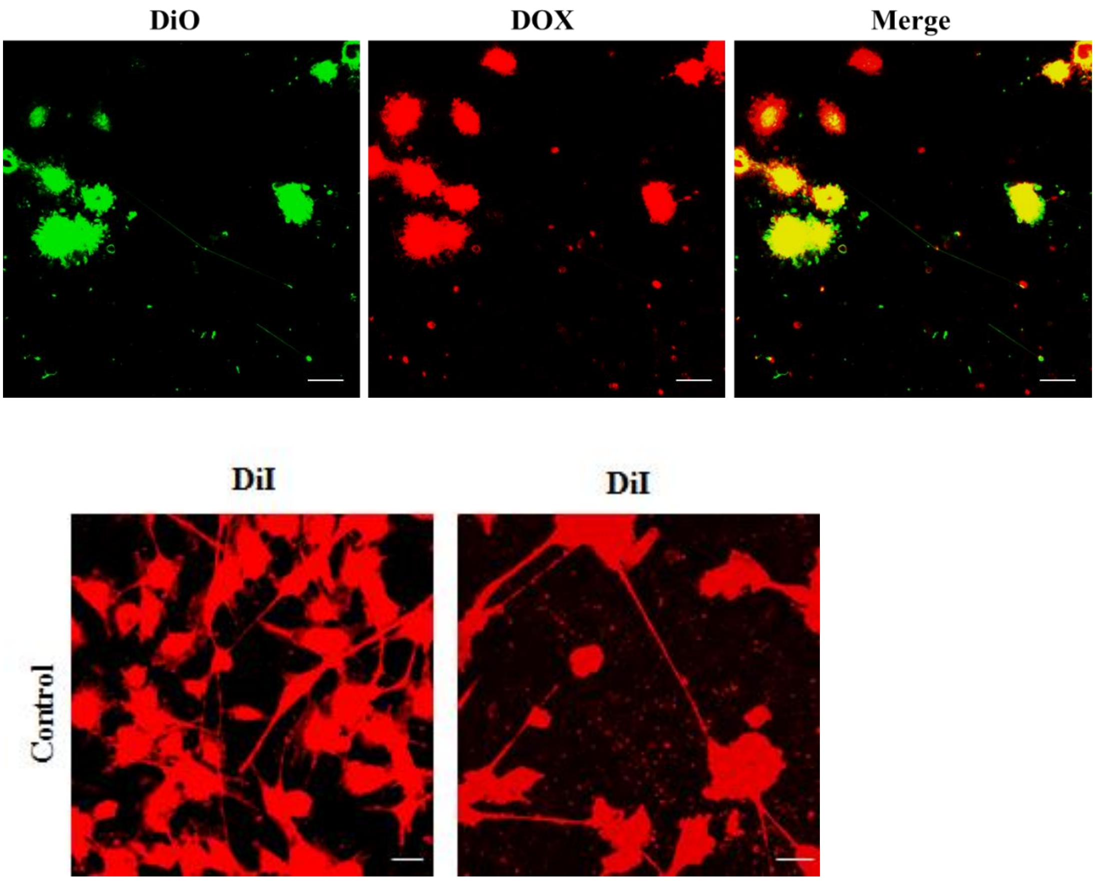

Figure S5. Free doxorubicin induces the TnTs disappearance and death of U87-GM cells. U87-MG cells were plated on gelatin pre-treated coverslips. Cells were leaved in culture complete medium for 48 h and then incubated with 15 µg/ml of free DOX for 24 h. Cells were later stained for 20 min with DiO (5 µl/ml) or DiI (5µM), fixed and fluorescence images were captured with a 40x magnification on A1R Nikon laser scanning confocal microscope. Scale bar: 10µm. DOX = doxorubicin.

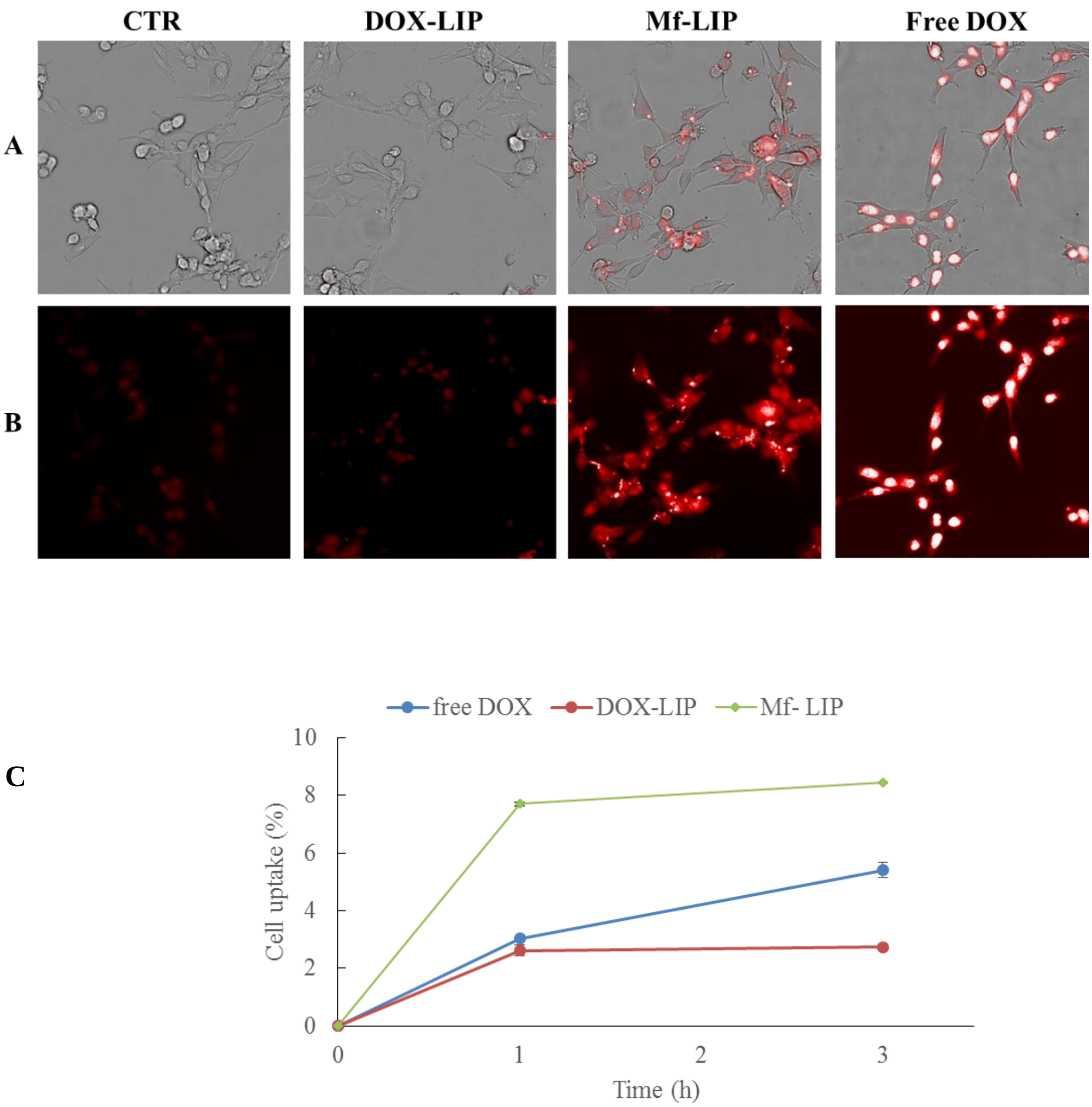

Figure S6. Cellular uptake of DOX by U87-MG cells. U87-MG cells were seeded on a 96-well plate (10^4 cells/well). After two days of culturing, cells were treated with free DOX (15 µg/ml) or DOX-LIP or Mf-LIP (15 µg/ml of DOX, 200 nmols of total lipids) and the uptake was evaluated by acquiring images at three different time points (0, 1 h, 3 h). Representative images at 1h are shown. Images were acquired in the brightfield channel (**A**) and in the DOX channel (λex=495 nm; λem=592 nm) (**B**) using a 40x air objective lens and standard instrument filters. Time course DOX uptake by U87-MG was measured by Jasco FP-8500 spectrofluorometer (**C**).

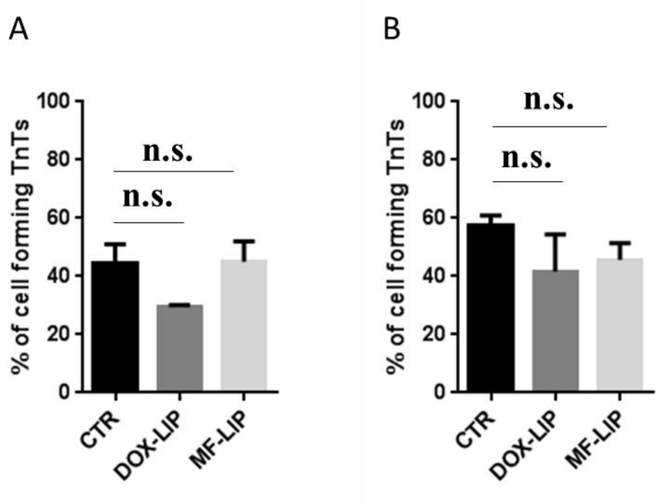

Figure S7. Percentage of cells forming TnTs after DOX-LIP or Mf-LIP treatment. Percentage of U87-MG cells (**A**) and NHA cells (**B**) forming TnTs on total cells is shown. At least 200 cells were analyzed per group in three independent experiments. Data are expressed as mean ± S.E from three independent experiments. Data were analyzed by one-way ANOVA followed by Dunnett's post-hoc test; n.s.= not significant. DOX-LIP = liposomes carrying doxorubicin; Mf-LIP = multi-functionalized liposomes; CTR = control untreated cells.
